# *Citrus × paradisi* L. Fruit Waste: The Impact of Eco-Friendly Extraction Techniques on the Phytochemical and Antioxidant Potential

**DOI:** 10.3390/nu15051276

**Published:** 2023-03-03

**Authors:** Jolita Stabrauskiene, Mindaugas Marksa, Liudas Ivanauskas, Pranas Viskelis, Jonas Viskelis, Jurga Bernatoniene

**Affiliations:** 1 Department of Drug Technology and Social Pharmacy, Lithuanian University of Health Sciences, LT-50161 Kaunas, Lithuania; 2 Institute of Pharmaceutical Technologies, Lithuanian University of Health Sciences, LT-50161 Kaunas, Lithuania; 3 Department of Analytical and Toxicological Chemistry, Lithuanian University of Health Sciences, LT-50161 Kaunas, Lithuania; 4 Lithuanian Research Centre for Agriculture and Forestry, Institute of Horticulture, LT-54333 Babtai, Lithuania

**Keywords:** *Citrus × paradisi* L., grapefruit, flavanones, aglycones, excipients, cyclodextrins, extractions, antioxidant

## Abstract

Citrus fruits have been the subject of extensive research over the years due to their impressive antioxidant properties, the health benefits of flavanones, and their potential use in the prevention and treatment of chronic diseases. Grapefruit have been shown in studies to improve overall health, with numerous potential benefits, including improved heart health, reduced risk of certain cancers, improved digestive health, and improved immune system function. The development of cyclodextrin complexes is an exciting approach to increasing the content of flavanones such as naringin and naringenin in the extraction medium while improving the profile of beneficial phenolic compounds and the antioxidant profile. This research aims to optimize the extraction conditions of the flavanones naringin and naringenin with additional compounds to increase their yield from different parts of grapefruit (*Citrus × paradisi* L.) fruits, such as albedo and segmental membranes. In addition, the total content of phenolic compounds, flavonoids, and the antioxidant activity of ethanolic extracts produced conventionally and with -cyclodextrin was examined and compared. In addition, antioxidant activity was measured using the radical scavenging activity assay (ABTS), radical scavenging activity assay (DPPH), and ferric reducing antioxidant power (FRAP) methods. The yield of naringin increased from 10.53 ± 0.52 mg/g to 45.56 ± 5.06 mg/g to 51.11 ± 7.63 mg/g of the segmental membrane when cyclodextrins (α, β-CD) were used; naringenin increased from 65.85 ± 10.96 μg/g to 91.19 ± 15.19 μg/g of the segmental membrane when cyclodextrins (α, β-CD) were used. Furthermore, the results showed that cyclodextrin-assisted extraction had a significant impact in significantly increasing the yield of flavanones from grapefruit. In addition, the process was more efficient and less expensive, resulting in higher yields of flavanones with a lower concentration of ethanol and effort. This shows that cyclodextrin-assisted extraction is an excellent method for extracting valuable compounds from grapefruit.

## 1. Introduction

**Figure 1:**
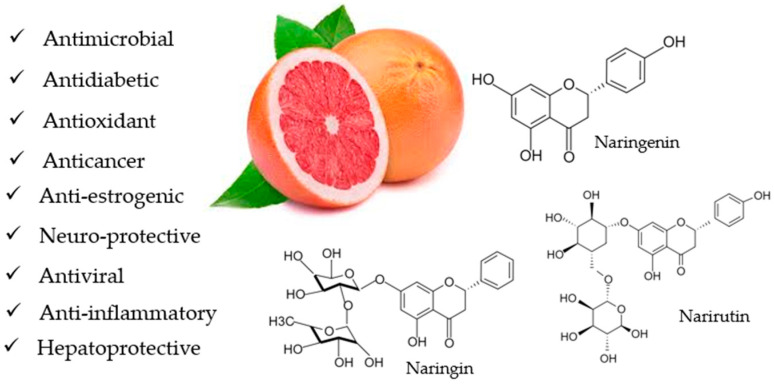
The effects of grapefruit (*Citrus × paradisi* L.) and the main flavonoids found in its extracts.

Natural bioactive compounds are in demand as humans become more health conscious, especially regarding a balanced diet. Epidemiological studies have shown that consumers of polyphenolic compounds are less susceptible to chronic diseases [1,2]. From this perspective, the fruits of *Citrus × paradisi* L. are rich in physiologically active components such as phenolic compounds, vitamins, carotenoid pigments, and fiber [3]. Grapefruit (*Citrus × paradisi* L.) is one of the world’s most popular fruits. In Eastern medicine, it is used as an appetite stimulant, antidiarrheal, emetic, and expectorant to treat flatulence, scurvy, acne, and eczema [4]. In addition, recent studies have shown that extracts of citrus peels and juices, as well as the biologically active components isolated from them, have a wide range of beneficial effects on living organisms, including antioxidant, antimicrobial, cardiovascular, anticancer, and antidiabetic activity (Figure 1) [5,6,7,8,9]. Due to the great pharmacological potential of citrus fruits, flavonoids and phenolic chemicals are the most studied biologically active molecules in the pharmaceutical field.

Two of the most important flavanones in grapefruit fruits are naringin and narirutin [10]. Naringenin (5,7,4′-trihydroxy flavanone), a polyphenolic flavonoid, is an aglycone derivative of hydrogenated flavone [11]. The bacteria of the gut microbiome convert naringin into active naringenin [12]. This flavonoid molecule is an essential part of the human diet and responsible for our foods’ color and bitter–sour taste.

The anticancer, antiproliferative, and antitumor effects of naringenin are based on its DNA repair ability [13,14]. It has inhibited breast, liver, prostate, melanoma, and spinal cord glioblastoma cells. Naringenin also influences the intrinsic (mitochondrial) and extrinsic (receptor) apoptotic pathways. The effect of naringenin on apoptosis inhibits proliferation and angiogenesis [15].

Naringenin reduces leukocyte accumulation by inhibiting macrophages’ chemotaxis molecules, which draw leukocytes to inflammation [14,16]. In addition, it activates NF-E2–related factor 2 (Nrf2), an anti-inflammatory factor, in macrophages which is another way it influences. It can also reduce pro-inflammatory cytokines such as IL-33, TNF-, IL-1, and IL-6, suppressing nuclear factor-κB (NF-ĸB) activation [17], boosting antioxidant ability, and reducing superoxide anions and other reactive oxygen species (ROS) [18].

Antidiabetic activity: Studies conducted in vitro and in vivo show that naringenin is essential in preventing and treating insulin resistance and type 2 diabetes [6]. This bioflavonoid can reduce the amount of glucose absorbed by the intestinal brush and the amount of sugar stored in the kidneys [19]. In addition, naringenin improves glucose uptake and utilization and contributes to glucose reabsorption by muscle and adipose tissue. According to the research, naringenin stimulates the growth of pancreatic cells, which has a beneficial effect. These cells have enhanced glucose-sensing abilities because of their training. It has been hypothesized that naringenin causes pancreatic cells to be more sensitive to the effects of glucose and has a pro-apoptotic effect on these cells [12].

Naringenin reduces inflammation caused by phenyl-β-benzoquinone, acetic acid, formalin, capsaicin, carrageenan, and superoxide anions [20]. This bioflavonoid also possesses antinociceptive and analgesic properties in vivo. Naringenin also regulates transient receptor potential (TRP) channels in nociceptors. Hence, it helps in analgesia [21].

Antibiotic resistance is a global problem. Bacteria are resistant to all groups of antibiotics [22]. The use of antibiotics in the food, veterinary, and medical industries has raised this concern. Overprescribing antibiotics to asymptomatic patients, the COVID-19 pandemic, and broad-spectrum antibiotics have worsened this situation. *Acinetobacter Baumann*, vancomycin-resistant *Enterococcus faecalis*, methicillin-resistant *Staphylococcus aureus*, and beta-lactam-resistant *Klebsiella pneumonia* result from overuse. Naringenin kills Gram-positive bacteria, including *S. aureus* and *MRSA* [23,24]. However, only a few clinical studies are using this bioflavonoid as an antibiotic. Unfortunately, neither the US nor the EU databases have registered any clinical trials, so these results are unavailable. Pharmacological safety is shown at 900 mg [25].

Flavanones are also liver protective. Naringenin reduces hyperglycemia, hyperlipidemia, and gluconeogenesis [26], reduces triglyceride formation, and significantly reduces low-density lipoproteins (LDL) and triglycerides in diabetic mice. Furthermore, based on articles, naringenin increased high-density lipoproteins (HDL) levels in Wistar albino rats [19,27].

**Figure 2:**
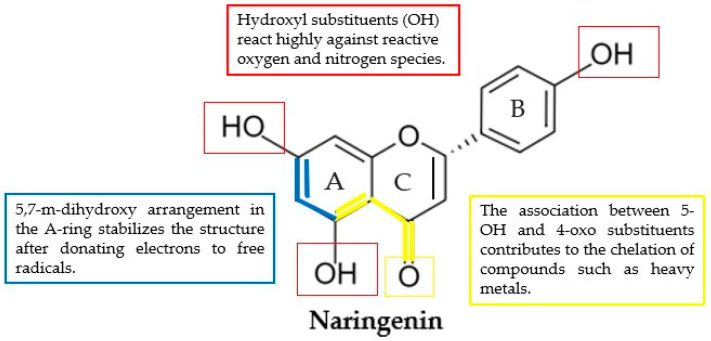
Naringenin: the relationship between antioxidant activity and structure. In red: Hydroxyl substituents (OH) react highly against reactive oxygen and nitrogen species. In blue: 5,7-m-dihydroxy arrangement in the A-ring stabilizes the structure after donating electrons to free radicals. In yellow: The association between 5-OH and 4-oxo substituents contributes to the chelation of compounds such as heavy metals.

Antioxidant activity in the traditional sense is identified by the hydroxy substituents (OH) on their molecules showing a high reactivity towards reactive oxygen species (ROS) and reactive nitrogen species (RNS) [5,22,28]. Because of this, the ability of a given molecule to function as an antioxidant increases when that molecule contains OH radicals; in the case of naringenin, there are three of these residues. After this, OH donates its hydrogen to free radicals (R), eventually stabilizing naringenin by resonance. Ring B is an essential part of the typical structure of flavonoids. This is because when hydroxyl groups are present in the ring, flavonoids can stabilize hydroxyl (OH), peroxyl (ROO), and peroxynitrite (ONOO) radicals, thereby forming relatively stable flavonoid radicals (Figure 2) [29,30].

UV spectrophotometry is the most common method for measuring phenolic compounds, flavonoids, and phenolic acids [31]. They measure the absorption of the reaction mixture in the visible spectrum. These methods are fast, simple, and reliable but lack chromatographic selectivity. Colorimetric techniques are used to quantify phenolic compounds based on their abundance and the complexity of the plant matrix [32]. One of these methods is that of Folin–Ciocalteu, which uses a specific reagent composed of several chemicals (sodium molybdate, sodium tungstate, etc.) [33]. The method is based on electron transfer reactions. However, the method requires a reference substance (in this case, gallic acid) to measure the total phenolic content of the extract.

Next, the method with aluminum chloride (AlCl_3_) is used to test the total amount of flavonoids. These forms chelate complexes of aluminum and flavonoids [34]. DPPH (2,2-diphenyl1-picrylhydrazyl), ABTS (2,2′-azino-bis(3-ethylbenzothiazoline-6-sulfonic acid), and FRAP (Ferric Reducing Antioxidant Power) are spectrophotometric techniques for the preliminary testing of the antioxidant activity of plant extracts [35,36].

ABTS uses ABTS•+, a stable blue-green radical with a maximum absorbance of 734 nm. Before use, allow the ABTS solution and potassium persulfate (K_2_S_2_O_8_) to react in the dark at room temperature for 12–16 h to generate the ABTS radical cation. Next, this ion reacts with phenolic chemicals to change the blue color to greenish or colorless. Data are Trolox equivalents [34].

The FRAP method is comparable to the ABTS and DPPH methods; however, the reaction is carried out in an acidic medium. The effectiveness of the antioxidant in reducing Fe (III) in an acidic solution is the focus of this approach [35].

Pharmacologically active chemicals usually contain excipients. Excipients help in the formulation of drugs, and they increase drug stability, dosage uniformity, and bioavailability [36]. Cyclodextrins are composed of a ring of glucose molecules linked in a specific arrangement. The structure creates a cavity within the molecule, which can be used to bind to other molecules. This property makes cyclodextrins useful for drug delivery, as they can bind to drugs and deliver them to the desired location in the body. Additionally, cyclodextrins have a high degree of solubility in water, which makes them useful for water purification applications.

Another property of cyclodextrins is their ability to form complexes with other molecules. This property makes them useful for food processing applications, as they can be used to bind to food additives and other compounds to improve their stability and shelf life. Additionally, cyclodextrins can be used to bind to flavors and aromas, which can be used to enhance the flavor and aroma of food products.

**Figure 3:**
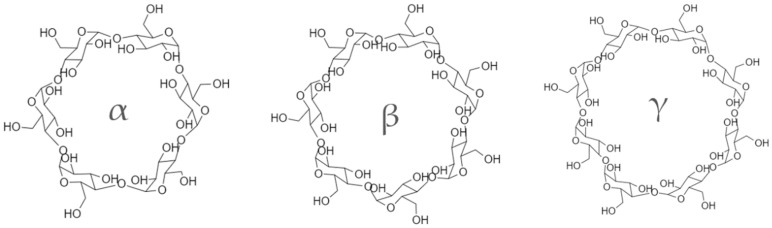
Chemical formulas of α-, β-, and γ-CD.

Cyclodextrins (CDs) form inclusion complexes in aqueous solutions, making them suitable for assisted extraction. Lipophilic guest molecules or fragments reside within these inclusion complexes [37], so complexation can increase flavanone solubility and antioxidant properties [38]. In addition, CDs decrease the taste of naringin generations and decrease their interaction with intestinal CYP3A4 metabolized drugs [39]. The most common types of cyclodextrins are α-CD, β-CD, and γ-CD, which contain six, seven, and eight glucopyranose units, respectively (Figure 3).

**Figure 4:**
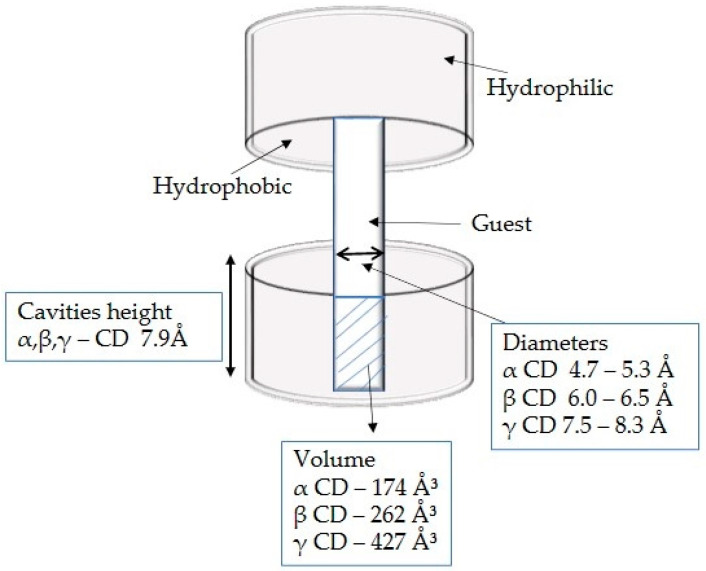
The heights, internal diameters, and cavity volumes of α, β, and γ CDs.

The exterior of these molecules is hydrophilic, which helps CDs interact favorably with water. Furthermore, the hydrophobic cavity of these molecules can accommodate a variety of guest molecules, including polar compounds (alcohols, acids, amines, and small inorganic anions) and nonpolar compounds (aliphatic and aromatic hydrocarbons) [40,41]. Figure 4 shows a graphical representation of the structure of CDs.

Recently, CDs have been used to separate polyphenols, including phenolic acid and flavonoids, from various natural sources. For example, Li Cui et al. (2012) found that β-CD inclusion complexation increased the solubility of naringin in water, which accelerated enzyme hydrolysis to form naringenin [42,43]. In addition, the inclusion complex foundation protects CDs from oxidation and decay [44].

Based on these assessments, it becomes clear why there is so much interest in conducting additional in-depth studies on treated grapefruit peel as a natural, economical, and accessible antioxidant source.

The purpose of this research is twofold: (I) to describe and optimize the extraction conditions of the flavanones naringin and naringenin with additional compounds to increase their yield from different parts of the grapefruit fruit, such as albedo and segmental membranes; and (II) to describe and quantify the flavonoid profiles and antioxidant activity of processed grapefruit peel and juice. Both objectives are addressed in this study.

## 2. Materials and Methods

### 2.1. Material

**Figure 5:**
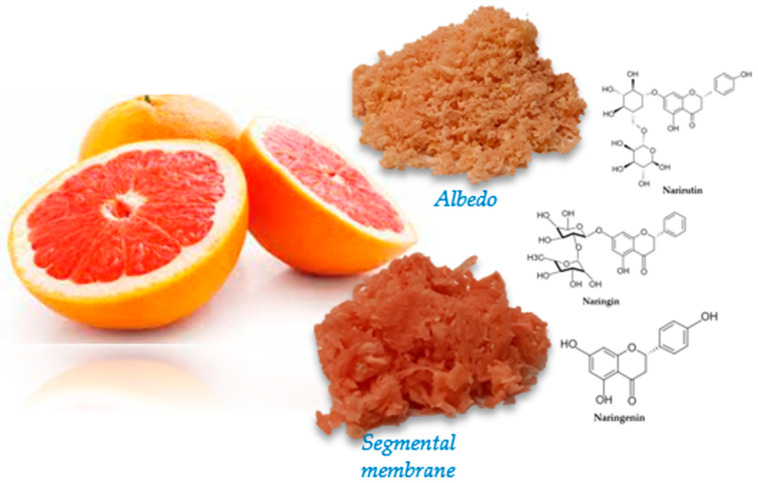
Fresh Albedo, and segmental membrane of *Citrus × paradisi* L.

The grapefruit’s segmental membranes and skins were gathered for secondary raw materials when the juice was extracted from grapefruit (*Citrus × paradisi* L., variety Star Ruby, Italy, unknown place). After being diced up in a food processor, these components were frozen at −18 ± 0.9 °C until extraction. Figure 5 depicts the fruit slices that were utilized in this investigation.

Standards of naringin, naringenin, and narirutin were obtained from Sigma Aldrich in Steinheim, Germany. Hydrochloric acid, sodium hydroxide, acetic acid, methanol, acetonitrile, Trolox, α-, β-, and γ-CDs were obtained from Sigma Aldrich in HH, DE. Ethanol (96%) was obtained from Vilniaus Degtine in Vilniaus, LT. GFL2004 was used to manufacture filtered water (Burgwedelis, DE). The following reagents were also utilized: aluminum chloride, hexamethylenetetramine, acetic acid, 2,20-azino-bis(3-ethylbenzothiazoline-6-sulfonic acid) (ABTS), 2,4,6-Tris(2-pyridyl)-s-triazine (TPTZ), potassium persulfate, ferrous sulfate heptahydrate, saline phosphate buffer, and hydrogen peroxide from Sigma Aldrich (Schnelldorf, Germany); disodium hydrogen phosphate obtained from Merck (Darmstadt, Germany); 2,2-diphenyl1-picrylhydrazyl radical (DPPH).

### 2.2. Methods

#### 2.2.1. Extracts’ Preparation

**Figure 6:**
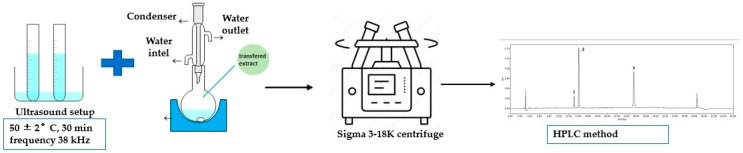
Combined extraction method schema.

Control sample: a control batch was performed based on previous studies using a combined extraction method with 50 and 70% ethanol (*v*/*v*). The extraction process used in this study is shown in Figure 6 [45].

Test sample: The extracts were made under the same conditions. The 50% or 70% ethanol (*v*/*v*) was employed as the solvent (10 mL), and the additional substances (0.1 ± 0.105 g of α-, β-, γ-CDs) were added with plant material (1 ± 0.05 g). After centrifuging the materials for 10 min at 1789× *g*, the supernatant was discarded by decantation. The extracts were then filtered using PVDF syringe filters with a pore size of 0.22 m, and an HPLC analysis was used to determine the total quantity of flavanones. A list of samples prepared in the UAE with thermal hydrolysis and excipients is given in Table 1.

#### 2.2.2. HPLC–PDA Conditions

**Figure 7:**
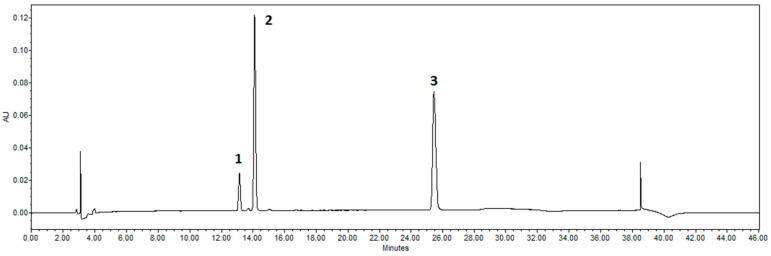
Chromatograms of standards detected by HPLC. 1–narirutin, 2–naringin, and 3–naringenin were identified as peak.

Waters 2695 liquid chromatography with a photodiode array detector was used (Waters 996, 200–400 nm wavelength range). We used an ACE C18 chromatography column (250 mm × 4.6 mm) with a sorbent particle size of 5 μm to separate physiologically active substances. The process details of the HPLC method were as follows. Gradient elution was used to separate the tested substances. Each extract was injected in a volume of 10 L and measured at 280 nm. Eluent A: acetonitrile at a rate of 1 mL/min; Eluent B: water. 0.0 min, 10% A; 5 min, 20% A; 25 min, 40% A; 30 min, 100% A; 35 min, 100% A; 36 min, 10% A. The temperature of the column was set at 25 °C. Peaks were found by comparing the UV-Vis spectra of each peak to valid reference standards and measuring their retention times. The samples were subjected to two different analyses. The chromatograms of the standards for naringenin, naringin, and narirutin are shown in Figure 7.

The Methodological Review of Natural Products by Wolfender (2009) [29,46] was used for quantification and validation. Standard stock solutions with primary concentrations of 100 µg/mL of naringin, narirutin, and naringenin were prepared in 70% methanol, and calibration curves were constructed using six standard solution concentrations. Three injections per concentration were performed to determine linearity. To construct calibration equations, naringin, naringenin, and narirutin were plotted against known concentrations of the respective standard solutions. The least squares approach was used to calculate a linear regression equation. The regression coefficients of all calibration curves were *R*^2^ > 0.999, confirming the linearity of the concentration ranges.

The sensitivity of the approach was determined by determining the limit of detection (LOD) and the quantification (LOQ). The concentrations that produced a signal-to-noise ratio of 3 and 10, respectively, were used to determine LOD and LOQ.

A standard mixture of naringin, naringenin, and narirutin was used during the intraday and inter-day precision testing. Five repeated non-consecutive injections of the regular combination on the same day on four different days proved the method’s accuracy. Relative standard deviation (RSD) is used to describe the results. The retention times and spectra of standards (naringin, naringenin, and narirutin) were compared to those prepared in extracts in this work. Linearity was determined by calculating the correlation coefficient *R*^2^ of the calibration curve (naringin *R*^2^ = 0.99992, naringenin *R*^2^ = 0.99992, narirutin *R*^2^ = 0.99999) and the peak areas were used for quantification. The linearity range of naringin was 1.166 to 33.34 μg/mL, 0.472 to 15.12 μg/mL for naringenin, and 1.2757 to 80.5 μg/mL for narirutin. The concentrations of naringenin, naringin, and narirutin were expressed as µg/g, mg/g, and mg/g dry weight (DW), respectively (Table 2).

### 2.3. Statistical Data Analysis

SSPS version 20.0 (IBM Corporation, Armonk, NY, USA) was used to analyze the data. Data are presented as mean and standard deviation (SD). All quantitative data were performed in triplicate. The Friedman and Wilcoxon tests were used to make the comparisons that should be made between the three different metrics. In addition, Spearman’s test was used to determine correlation and regression coefficients. At the end, a comparison was made between the two groups using the Mann–Whitney U test. Results were considered statistically significant (*p* < 0.05).

### 2.4. Preparation of Extracts for Total Phenolic, Flavonoid, and Antioxidant Activity

**Figure 8:**
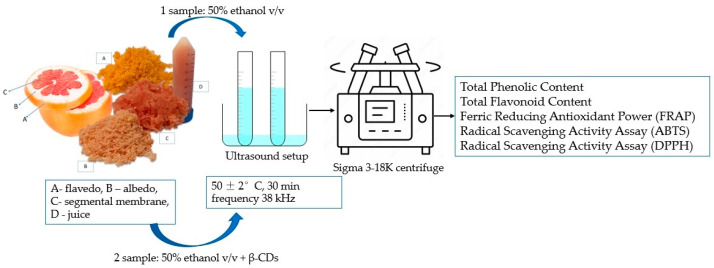
Extraction procedure to determine total phenolic, flavonoid, and antioxidant activity.

We used a modification of this ultrasound-assisted extraction method to adapt it for our application to determine the total phenolic, flavonoid amount, and antioxidant activity. An ultrasound-assisted extraction method (UP-250; frequency range: 19–25 kHz, 250 W, probe amplitude: 35 μm) was used to prepare the extraction from grapefruit waste product and fresh juice. Control sample: the total content of phenols, flavonoids, DPPH, ABTS, and FRAP was determined using the flavedo, albedo, segmental membrane. A total of 10 g of plant material was poured with a solvent (50% ethanol *v*/*v*) and treated with an ultrasonic bath at 50 ± 5 °C for 30 min. The tested sample was produced under the same conditions but with additional excipients (0.1 g ± 0.01 g β-CDs) (Figure 8). A fresh juice tested sample was created; 10 mL of fresh juice and 0.1 g ± 0.01 g of β-cyclodextrins were added and treated for 30 min at 50 ± 5 °C using an ultrasound bath.

#### 2.4.1. Analysis of Total Phenolic Content

A spectrophotometric analysis method determined the total amount of phenolic compounds in *Citrus × paradisi* L. The extract was mixed with the Folin-Ciocalteu phenol reagent, 2 mL of 7% sodium carbonate (Na_2_CO₃) was added, and the mixture was kept in a dark place for 60 min. A gallic acid calibration curve y = 12.069x; *R*^2^ = 0.9978 was used to create the calibration curve. The data were given in milligrams of gallic acid equivalent per gram of dry weight (mg GAE/g DW) [47].

#### 2.4.2. Total Flavonoid Content Evaluation

The colorimetric aluminum chloride technique was modified to determine the total flavonoid concentration of the extracts [48]. First, 0.2 mL of the extract was combined with 2 mL of 96% (*v*/*v*) ethanol, 0.1 mL of 30% acetic acid, 0.3 mL of 10% aluminum chloride (AlCl₃), and 0.4 mL of 5% hexamethylenetetramine solutions. After 30 min of incubation, the absorbance of the reaction mixture was measured at 475 nm using a spectrophotometer (Shimadzu UV-1800; Kyoto, Japan). Then, the total flavonoid content was determined using a calibration curve (y = 5.0867x; *R*^2^ = 0.9985). The result was computed using the following procedure and expressed as mg of rutin equivalent per gram of dry weight (RE/g DW):

TFC = C × Ve × F/m,

where

TFC–total flavonoid content; mg RE/g DW;
C–concentration of standards used mg/L;
Ve–the volume of solvent used;
F–dilution coefficient of the sample; m–a mass of the sample, g.

### 2.5. Antioxidant Activity

#### 2.5.1. Radical-Scavenging Assay (ABTS)

ABTS (2,2′-Azino-bis-(3-ethylbenzthiazoline-6-sulonic acid) was oxidized using potassium persulfate to generate the ABTS•⁺ radical. It showed absorption maxima at wavelengths 645, 734, and 815 nm. A 7 mM (ABTS) aqueous solution was created and stored in the dark for 12 to 16 h to generate a dark solution containing the radical cation. Before usage, the ABTS radical cation was diluted with water, and its initial absorbance at 734 nm was measured using a spectrophotometer to be about 0.70 ± 0.01. To assess radical scavenging activity, 2.0 mL of the ABTS working standard was combined with 200 µL in a test cuvette. Using Trolox, the calibration curve was created (y = 0.0001728x; *R*^2^ = 0.9832). Results were reported regarding the Trolox equivalent per gram of dry weight (TE per g DW) [49].

#### 2.5.2. Radical Scavenging Assay (DPPH)

The free radical scavenging activity of the extract was carried out with slight modifications. For the first step, 10 μL of each ethanolic solution was mixed with 3 mL of 2,2-diphenyl-1-picrylhydrazyl (DPPH) solution. Then, the reaction mixture was incubated in the dark at room temperature for 1 h, and the absorbance at 517 nm was measured using a spectrophotometer (Shimadzu UV-1800; Kyoto, Japan). The calibration curve was obtained with Trolox (y = 0.00623x; *R*^2^ = 0.9923). The results were expressed as Trolox equivalent per gram dry weight (TE/g DW) [50].

#### 2.5.3. Ferric Reducing Antioxidant Power (FRAP)

The FRAP test was performed by combining 0.3 M of acetate buffer with a pH of 3.6, 10 mM of a solution containing 2,4,6-tripiridil-s-triazino, 40 mM of hydrochloric acid, and 20 mM of a solution containing ferric (III) chloride (10:1:1). After that, 10 µL of the sample was combined with 3 mL of the FRAP reagent, and the resulting mixture was well combined. After 30 min incubation, using a spectrophotometer, the level of absorption was determined to be 593 nm (Shimadzu UV-1800; Kyoto, Japan). Ferrous sulfate was used to obtain the calibration curve, which had the following equation: y = 0.00010x + 0.0646; *R*^2^ = 0.9915 [51].

## 3. Results and Discussion

The flavanones most prevalent in grapefruit fruit are glycosides, specifically naringin and narirutin. On the other hand, naringenin (in the form of aglycone) is not very soluble in water; as a result, the majority of the extracts that were tested either had very low amounts of aglycone or only traces of it [42]. Consequently, one of the essential tasks is to increase the amount of naringenin in the extracts and to do so in the simplest, most viable way. We selected the most optimal extraction conditions based on the previous research methods and used the conjugated extraction method. Using only ultrasound extraction method (UAE) without thermal hydrolysis showed poor results compared with thermal hydrolysis. Thermal hydrolysis is necessary to obtain a higher yield of naringenin (Table 3).

First, the control extraction with the α, β, and γ-CDs was performed using the conjugated extraction method described in Section 2.2.1 and compared to the test extraction (prepared under the same conditions). Table 3 illustrates the findings that were obtained from the control extraction.

### 3.1. The Quantity of Flavanones Using the Additional Substances α-, β-, γ-Cyclodextrins

The structure of cyclodextrins (CD) can explain the increased yield of flavanones in the extracts from plant material. Additionally, the CD encapsulation can change the physicochemical features, such as water solubility and the substance’s stability [36].

CDs can protect chemically unstable drug molecules from environmental factors, reducing or preventing drug hydrolysis, oxidation, and enzymatic degradation. One common application is reducing bitterness in citrus fruit juice by forming a naringin-β-CD complex [38]. The research of Pereira et al. in 2021 revealed that by using inclusion complexes, an impressive conversion rate from naringin to naringenin could be achieved, reaching up to 98.7% and 56.2%. On top of this remarkable success, β-CD has been noted as having a positive effect on water solubility; therefore, enzymatic hydrolysis of naringin will likely improve significantly [44].

Based on previous studies, we picked the albedo and segmental membrane parts for our investigation. The most significant quantities of naringenin were detected using combination extraction methods (ultrasonic extraction method with thermal hydrolysis).

Due to its polarity for flavonoids, research shows that methanol, among other organic solvents, is exceptionally capable of extraction. Because of this, efforts are being undertaken to find non-toxic and entirely biodegradable alternatives, such as ethanol, that can produce the same or similar outcomes without harming the environment [52,53].

The primary goal was to improve the flavanone yield in the extract; an organic solvent was chosen as a co-solvent with CDs because aglycones are more hydrophobic than hydrophilic chemicals and have limited solubility in water. As a result, once the combination with CD degrades, flavanone deposits might develop in water. This procedure may be avoided by using an organic solvent.

In control, the maximum level of naringenin was detected in the part of the segmental membrane using 70% ethanol (67.59 ± 3.37 µg/g). By using cyclodextrins, this amount could be increased by more than 34%. Comparing α, β, and γ-cyclodextrins, a statistically significant increase was observed in the AA1 sample (α-CDS, 50% ethanol *v*/*v*) 43.44 ± 2.17 µg/g compared to AT1 (23.58 ± 1.17 µg/g), AB1 (16.21 ± 0.81 µg/g), AG1 (0.84 ± 0.042 µg/g), and SA1 (91.19 ± 4.55 µg/g) compared to ST1 (65.84 ± 3.29 µg/g) and SB1 (0.45 ± 0.02 µg/g).

The results of our samples concluded that the combination of naringenin and α-CD with the smallest cavity size provided a higher yield of the aglycone compared to other cyclodextrins. Statistical analysis indicated that this significant increase was remarkable (*p* < 0.05).

**Figure 9:**
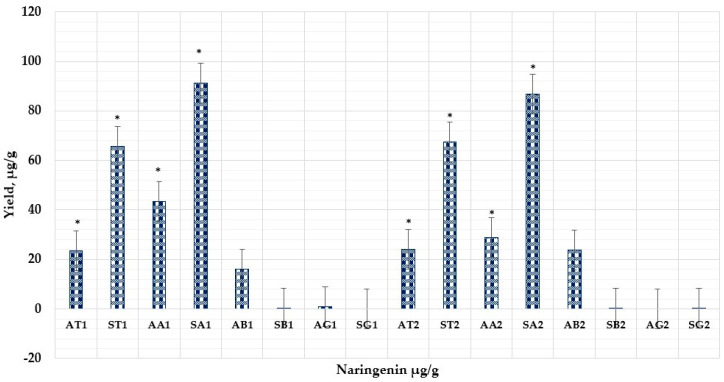
The quantitative yield of naringenin using excipients (1%). First letter demonstrated fruit parts (A—lbedo; S—segmental membrane); second letter (T—control sample; A—α-CD; B—β-CD; G—γ-CD); numbers (1–50% solvent *v*/*v*; 2–70% solvent *v*/*v*). AA1 *p* < 0.05 vs. AT1; AA2 *p* < 0.05 vs. AT2; SA1 *p* < 0.05 vs. ST1; SA2 *p* < 0.05 vs. ST2; AA1 *p* < 0.05 vs. AA2; AB2 *p* < 0.05 vs. AB1. * Results are means ± SD (*n* = 3).

According to the results of our research, 50% ethanol generated a higher concentration of naringenin than 70% (*v*/*v*) ethanol using α-CD: (AA1) 43.44 ± 2.17 µg/g–(AA2) 28.77 ± 1.44 µg/g from albedo, and (SA1) 91.19 ± 4.55 µg/g–(SA2) 86.69 ± 4.33 µg/g from segmental membrane parts, (50% and 70%, respectively). In addition, control samples AT1, AT2, and ST1 and ST2 were compared to test samples using α, β, and γ-CDs yielding statistically significant findings for naringenin using α-CD. The quantitative yield of naringenin using excipients (1%) is shown in Figure 9.

**Figure 10:**
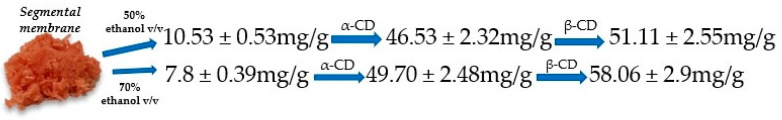
Naringin yield (control sample < α-CD < β-CD, respectively). Results are means ± SD (*n* = 3).

Both naringin and narirutin are examples of hydrophobic polyphenols with a low water solubility of 38 and 500 µg/mL, respectively, at room temperature [54]. In addition, these polyphenols are inherently polar due to at least one sugar moiety in their molecular structures [55]. They are unable to form a stable complex with α-CD because they have the lowest affinity for it. In contrast to the findings obtained with control samples, extracts obtained with α-CD in this investigation showed statistically significant improvement (Figure 10) (*p* < 0.05). The results of flavanones showed in Table 4.

Based on the literature and previous research, our study chose 50 or 70% ethanol *v*/*v*. The extraction with water did not show any meaningful results, even after adding additional substances. Meanwhile, using 50% ethanol and α-CD significantly increased the amount of aglycones from the albedo and segmental membrane parts by an average of 1.6 and 1.05. Naringin from the albedo part (18.87 ± 0.94 mg/g–11.58 ± 0.58 mg/g, 50–70%, respectively) and narirutin in the sample from the albedo part 3.33 ± 0.16 mg/g–2.25 ± 0.11 mg/g, 50–70%, respectively.

**Figure 11:**
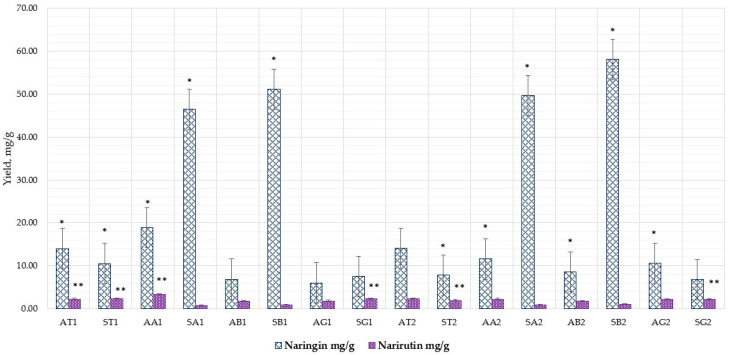
The quantitative yield of naringin and naringenin using excipients (1%). Sample codes are provided in Table 1. Results are means ± SD (*n* = 3). Naringin *: AA1 *p* < 0.05 vs. AT1 *; SA1 * *p* < 0.05 vs. ST1 *; SB1 * *p* < 0.05 vs. ST1 *; SB1 * *p* < 0.05 vs. SA1 *; AA2 *p* < 0.05 vs. AB2; SB2 * *p* < 0.05 vs. ST2 *; SB2 * *p* < 0.05 vs. SA2 *; SB2 * *p* < 0.05 vs. SB1 *; SA2 * *p* < 0.05 vs. SA1 *. Narirutin **: AA1 ** *p* < 0.05 vs. AT1 **; SG1 ** *p* < 0.05 vs. ST1 **; SG2 ** *p* < 0.05 vs. ST2 **.

The results of this study demonstrate that the quantity of flavanone in the extracts examined was significantly elevated when α and β cyclodextrins were used as compared to extracts used as controls. This study also showed that CDs increased flavanone yield while requiring a lower solvent concentration. Figure 11 displays the quantitative yield of naringin and naringenin when using excipients at a concentration of 1%.

The amount of naringin increased statistically significantly in the test sample with β-CDs, AA1, SA1, SA2, and SB2 compared to the control group AT1, ST1 AT2, and ST2. In addition, when comparing the test samples, higher amounts of naringin were found in the segmental membrane with β-CD using solvent 70% ethanol *v*/*v* (SB2) 58.08 ± 2.90 mg/g versus SB1 (50% ethanol *v*/*v*) 51.11 ± 2.55 mg/g.

The amount of narirutin was increased by α-CD in the albedo part, compared to the control sample (3.33 ± 0.16 mg/g vs. 2.24 ± 0.12 mg/g) and slightly increased by γ-CD in the segmental membrane 2.15 ± 0.11 mg/g). For naringin, the best results were obtained with β-CD from segmental membrane, using solvent 70% ethanol *v*/*v*.

### 3.2. Total Phenolic and Flavonoid Content Determination

It was evaluated how many total phenolic and flavonoids were determined using the ultrasound assistant extraction method (control sample) versus conjunction with 1% β-cyclodextrins (CDs) (tested sample), as described in Section 2.4.

Many medicinal plant species exhibit interspecific chemical diversity, which is important to study and evaluate. Chemical diversity studies provide information on the active ingredient’s composition across species, varieties, and plant parts. Because of this, using UV–visible light spectrophotometry, pilot tests were carried out to measure the total content of phenolic compounds and flavonoids from various parts of *Citrus × paradisi* L. fruits. According to the findings of the tests, the total phenolic content varied from 2.48 ± 0.124 mg GAE/g DW (flavedo), 3.58 ± 0.17 mg GAE/g DW (albedo), 2.56 ± 0.12 mg GAE/g DW (segmental membrane), and 2.89 ± 0.14 mg GAE/g DW (juice). Most samples prepared with excipients contained more total phenolic compounds than control samples (*p* < 0.05).

**Figure 12:**
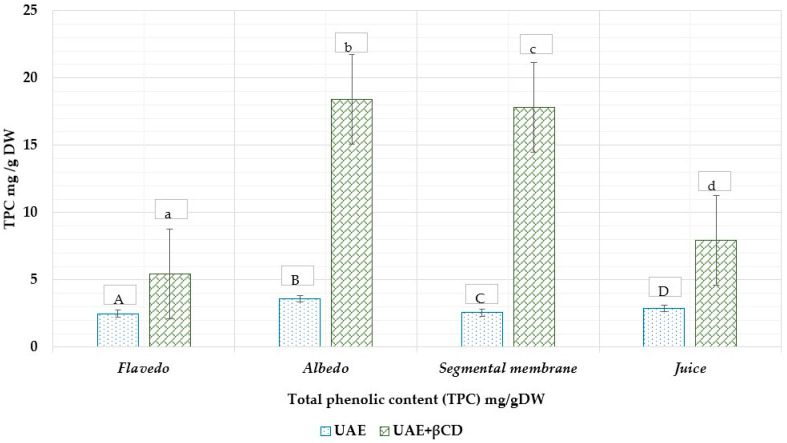
The total phenolic content TPC in grapefruit ethanolic extract with and without β-CDs. A, B, C, D—ultrasound extraction method (UAE), a, b, c, d—ultrasound extraction method (UAE) + β-CDs. The result is the mean value (*n* = 3) a, b, c, d *p* < 0.05 vs. A, B, C, D.

This finding is significant because it shows that excipients can enhance the number of phenolic compounds found in a sample and shows promise for future pharmacology developments. Furthermore, the fact that these results have been statistically significant at *p* ≤ 0.05 reinforces their credibility. Using β-CDs, the highest amount was found in the albedo part and increased from 3.58 ± 0.17 to 18.42 ± 0.92 mg GAE/g DW; the segmental membrane increased from 2.56 ± 0.128 to 17.8 ± 0.89 mg GAE/g DW. The total phenolic TPC content in grapefruit ethanol extract is shown in Figure 12.

According to Gorinsten et al., total phenolic content (TPC) content was significantly higher in fresh grapefruit peel (155 ± 10.3 mg/100 g) than in peeled grapefruits (135 ± 10.1 mg/100 g) [56]. The difference in total flavonoid content across studies might be attributed to differences in variety, location, or analytical methodologies [34]. Xi et al. observed that the total phenolic content varied by variety and fruit portion, varying from 3.17 to 4.63 mg/g GAE FW in the peel, 2.43 to 3.46 mg/g FW in the pulp, 0.29 to 0.52 mg/g FW in the juice, and 2.12 to 3.36 mg/g FW in the seeds [57]. According to the findings of the studies, the concentration of phenolic compounds present in albedo is higher than that found in any of the other portions of the fruit. This disparity may be attributable to the distinct parts of the fruit having a unique phenolic compound profile and a unique quantity of phenolic compounds. In addition, the solvent used to extract the substance, the method used to remove it, and the quality of the raw material might all contribute to the variations.

The concentration of flavonoids varies depending on the plant’s development stage since they are the most abundant group of chemicals found in plants and substantially influence the plant when growing. The majority of flavonoids, also known as flavanone glycosides, are only found in citrus trees. Other types of plants have very few of these compounds. Grapefruit contains several flavonoid glycosides, the most important of which are naringin, hesperidin, and narirutin [34,52].

Flavonoids are abundant in various parts of the fruit, resulting in significant differences between fruit types. Another study found that fruit parts and cultures had different total flavonoid levels. For example, the peel has values between 5.12 mg and 8.30 mg per gram fresh weight; the pulp can range from 3.86 to 5.38 mg/g, from 0.26 to 0.44 mg/g in juice, from 3.16 to 9.27 mg/g in whole fruits and from 18, 61 to 25.33 mg/g in seeds. As a result, seeds contained more flavonoids than juices (5496 times as much as the juice) [57].

Nurcholis et al. investigated the effects of extraction procedures and durations on total flavonoid and phenolic contents (TFCs and TPCs) in a solvent such as methanol. According to the researchers, different extraction procedures and durations significantly influenced the TFCs, TPCs, and antioxidant activities of Java cardamom fruit methanol extracts [58].

**Figure 13:**
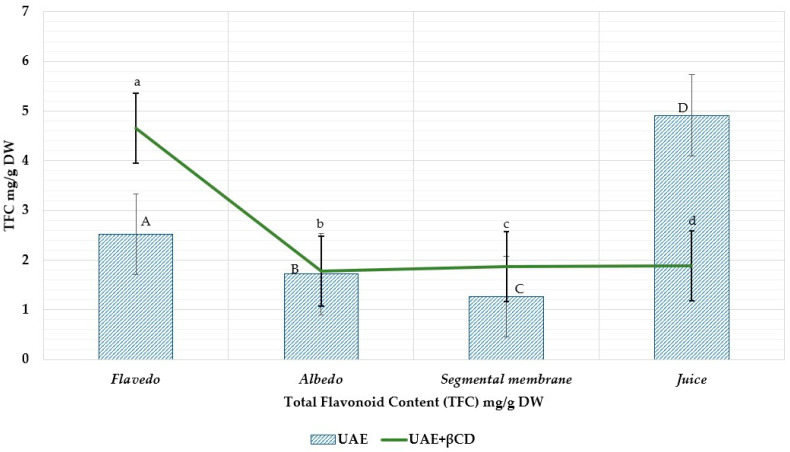
The total flavonoid content (TFC) in grapefruit ethanolic extract with and without β-CDs. A, B, C, D—ultrasound extraction method (UAE), a, b, c, d—ultrasound extraction method (UAE) + β-CDs. The result is the mean value (*n* = 3) a, b, c, d *p* < 0.05 vs. A, B, C, D.

Using the aluminum chloride technique, the flavonoid content of grapefruit fruit was analyzed, and the findings are presented in Figure 13. The TFC ranged from 1.26 ± 0.08 to 4.91 ± 0.24 mg RE/g DW. Flavedo extracts had the highest flavonoid levels (2.52 ± 0.13 mg RE/g DW). The total flavonoid content ranged from 1.78 ± 0.09 to 4.66 ± 0.23 mg RE/g DW using β-CDs using 50% ethanol (*v*/*v*) as a solvent. When comparing grapefruit extracts made under the same conditions but with an additional excipient, the total flavonoid content increases by 2.516 ± 0.1278 mg RE/g DW to 4.66 ± 1.26 mg RE/g DW in the flavedo parts. Meanwhile, using AlCl_3_ to determine the total flavonoids reduces the number of flavonoids from 4.91 ± 0.24 mg RE/g DW to 1.887 ± 0.094 mg RE/g DW in fresh juice.

### 3.3. Antioxidant Activity

After researching the total phenolic and flavonoid content of *Citrus × paradisi* L., the next step is to investigate and evaluate the antioxidant activity of the various parts of the fruit. The findings will be useful in evaluating and standardizing the quality of raw materials and their products. Furthermore, they will make it possible to anticipate the antioxidant activity of grapefruit extracts derived from various parts of the fruit when tested in vivo [59]. The antioxidant capacity of plant extracts can be affected by a wide variety of factors, including the extraction process, the solvent used, the kind of fruit, and the stage of ripeness at which the fruit was harvested [60]. Consequently, we decided to assess the number of antioxidants present in grapefruit using different methods (radical scavenging, antioxidant activity, and reducing power) [61].

Cyclodextrins, for example, can assist overcome the disadvantages of antioxidants in functional foods. In addition, cyclodextrins are also used as anti-browning agents to prevent the enzymatic browning of food. Finally, research shows that cyclodextrins act as secondary antioxidants and help typical antioxidants resist enzymatic browning.

Based on the literature review, the antioxidant capacity in the flavedo, albedo, segmental membrane, and fresh juice was determined. Extracts were extracted using ultrasound (as described in Section 2.4) (control test) and compared to extracts with the addition of β-cyclodextrins (test sample). To this end, the variation in antioxidant capacity was determined by assessing the effect of cyclodextrin on antioxidant capacity.

**Figure 14:**
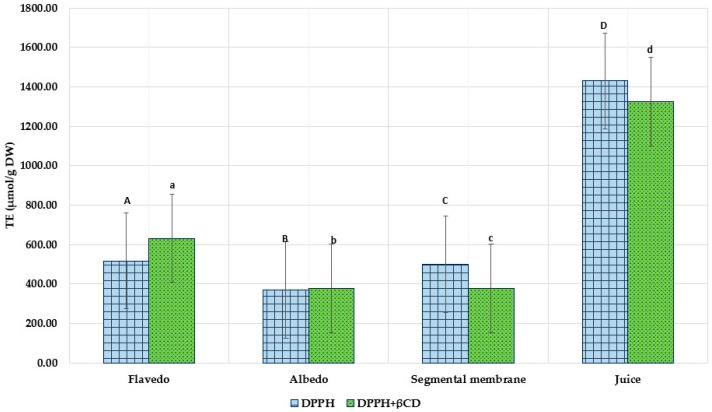
DPPH radical scavenging activity (DPPH) of ethanolic extracts with excipients and control sample (DPPH). Results are mean values (*n* = 3). A, B, C, D—ultrasound extraction method (UAE), a, b, c, d—ultrasound extraction method (UAE) + β-CDs. a, b *p* < 0.05 vs. A, B; a *p* < 0.05 b, c; D *p* < 0.05 A, B, C; d *p* < 0.05 a, b, c.

Figure 14 depicts the results of calculations made with DPPH to determine the radical-scavenging activity of grapefruit fruit in its various areas. After determining the antioxidant activity by the DPPH method, it was observed that the fresh juice and *flavedo* parts of the studied fruit neutralized the DPPH radical the most. The order of action was juice > flavedo > segmental membrane > albedo (1429.25 ± 71.01 μmol/g > 517.14 ± 25.86 μmol/g > 500.27 ± 22.54 μmol/g > 368.50 ± 15.42 μmol/g). β-CD was only slightly increased by DPPH radical inhibition in flavedo extracts from 517.14 ± 25.86 μmol TE/g DW to 630.76 ± 31.54 μmol TE/g DW. However, the CDs reduced the antioxidant activity in segmental membranes and juice.

The ABTS radical cation decolorization test is a method used to measure the antioxidant activity level of a substance. A solution of ABTS (2,2′-azino-bis (3-ethylbenzthiazoline-6-sulfonic acid)) and potassium persulfate is used. When these two compounds are mixed, they form a stable radical cation that can be used to measure the antioxidant activity of a substance. The radical cation ABTS is blue and can be decolorized by antioxidants. The extent of discoloration is proportional to the antioxidant activity of the substance to be tested [62].

The free radical scavenging activity of *Citrus × paradisi* L. extract varied considerably: from 4.36 ± 0.218 µg TE/g to 18.61 ± 0.93 µg TE/g. The highest ABTS radical-cation binding activity was observed in fresh grapefruit juice at 18.61 ± 0.93 µg TE/g.

**Figure 15:**
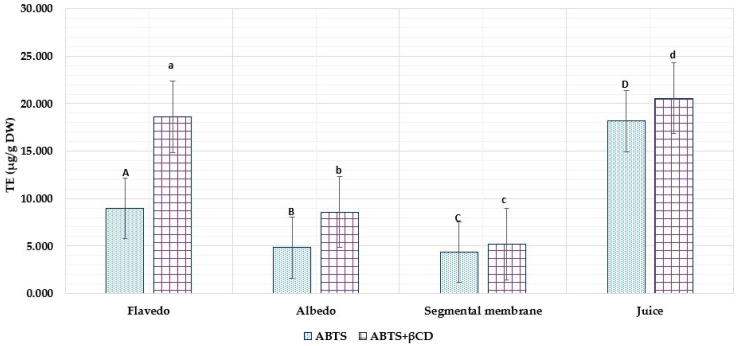
ABTS radical scavenging activity of ethanolic grapefruit extracts with excipients and without. The results are mean values (*n* = 3). All the test samples (a, b, c, d) were statistically (*p* < 0.05) significant to controls (A, B, C, D); D *p* < 0.05 A, B, C; d *p* < 0.05 a, b, c; A *p* < 0.05 B, C; a *p* < 0.05 b, c.

In this study, all test samples developed using CDs had significantly increased antioxidant activity than the control samples. In the sample with flavedo (from 8.97 ± 0.448 µg TE/g to 18.61 ± 0.93 µg TE/g) and fresh juice (from 18.61 ± 0.93 µg TE/g to 20.56 ± 1.028 µg TE/g), the highest binding of the ABTS radical showed (Figure 15). Conversely, the segmental membrane had the lowest antioxidant activity (4.36 ± 0.218 µg TE/g).

A compound’s ability to reduce other substances might be a valuable signal of its prospective antioxidant action. Combinations with reducing power imply that they are electron donors and can decrease the oxidized intermediates produced during the process of lipid peroxidation, functioning as both primary and secondary antioxidants [63].

It was discovered by Gupta et al., who investigated two distinct harvests of pomelo fruit, that the flavedo of the fruit had the highest antioxidant capacity and FRAP activity. On the other hand, the albedo of the fruit was found to have the highest accumulation of naringin. These findings were based on the findings that the flavedo of the fruit had the highest antioxidant capacity and FRAP activity. On the other hand, pomelo juice showed the most increased DPPH free-radical scavenging activity and the highest tannin concentration [59].

**Figure 16:**
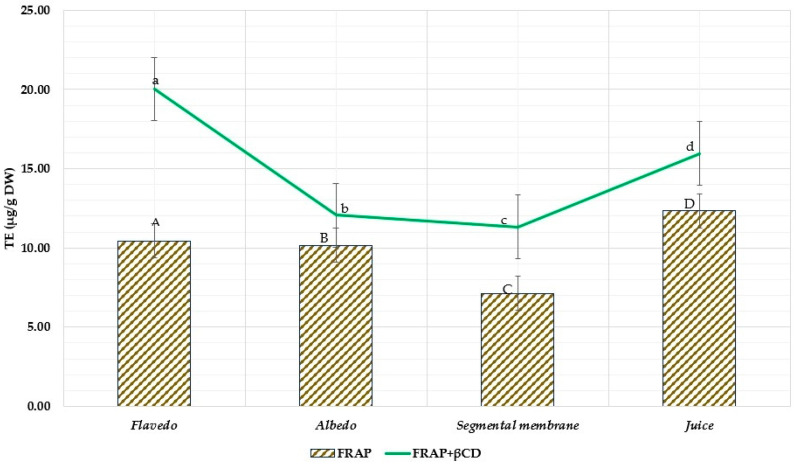
FRAP assay of ethanolic grapefruit extracts with excipients and without. The results are mean values (*n* = 3). All the test samples (a, b, c, d) were statistically (*p* < 0.05) significant to controls (A, B, C, D); D *p* < 0.05 A, B, C; a *p* < 0.05 b, c, d; d *p* < 0.05 c, d; A *p* < 0.05 B, C.

The results of a FRAP experiment that was conducted on various grapefruit components may be found in Figure 16. Compared to their capacity to scavenge free radicals, the ability of some samples to reduce iron ions (Fe^3^⁺) to iron ions (Fe^2^⁺) was significantly more impressive. Applying β-CD resulted in a one- to two-fold increase in the ethanol samples’ reduction power. In this investigation, the outcomes of the test samples (which had β-CDs) were noticeably superior to those of the control samples (devoid of β-CD).

The spectrophotometric FRAP method determined that fresh grapefruit juice and extracts from the flavedo showed the highest reducing activity among the tested extracts, and the lowest reducing activity was found in the segmental membrane. The use of β-cyclodextrins showed statistically significant results in all tested extracts. In some samples, the reductive activity doubled; for example, the sample from flavedo parts increased reduction from 10.44 ± 0.522 µg TE/g to 20.44 ± 1.02 µg TE/g. Figure 16 depicts the FRAP assay of ethanolic grapefruit extracts with and without excipients.

According to the findings of our research, the segmental membrane extract was the sample that possessed the least amount of antioxidant potential, as indicated by its TE values of 500.27 ± 25.89 μmol/g, 4359.57 ± 24.89 μmol/g, and 7136.00 ± 304.87 μmol/g for DPPH, ABTS, and FRAP, respectively. These numbers are expressed as standard errors. On the other hand, the fresh juice exhibited the highest level of antioxidant activity; the results for DPPH, ABTS, and FRAP were as follows: 1429.25 ± 90.86 μmol/g, 18152.01 ± 698.72 μmol/g, and 12336 ± 616.8 μmol/g, respectively. In addition, the extract from the flavedo part of the grapefruit showed significant results for both DPPH and ABTS (TE 517.14 ± 25.15 and 8969.91 ± 448.50 μmol/g, respectively), and the extracts from the albedo part showed strong FRAP reducing activity (TE 10169.33 ± 508.46 μmol/g).

## 4. Conclusions

In summary, waste derived from grapefruit can be recycled in the manufacture of future pharmaceutical and medical consumables. Therefore, the extraction of natural products and the research of natural goods and their possible uses are attracting increasing attention. However, to achieve the main goal, such as achieving higher yields of bioactive chemicals from natural sources in less time, new and safe, economically feasible, environmentally friendly extraction processes must first be created.

Cyclodextrins are a type of carbohydrate molecule that exhibit various unique properties that make them useful for various applications. These properties include their ability to form complexes with other molecules, their high solubility in water, and their ability to bind to drugs and other compounds. As a result, cyclodextrins have a variety of uses in the medical, food processing, and water purification industries and are essential tools in improving the safety and effectiveness of these industries.

The results of this study demonstrate that the quantity of flavanone in the extracts examined was significantly elevated when α and β cyclodextrins were used as compared to extracts used as controls. This study also showed that CDs increased flavanone yield while requiring a lower solvent concentration. The highest yield of naringenin was found in SA1, 91.19 ± 2.93 µg/g, prepared with α-CD and 50% ethanol *v*/*v*, and for naringin 58.68 ± 2.93 mg/g, prepared with β-CD and 70% ethanol *v*/*v*. The highest narirutin amount was observed in AA1 3.33 ± 0.166 mg/g using α-CD in 50% ethanol *v*/*v*. In contrast, γ-CD did not provide a statistically significant outcome in this investigation. In the culinary, nutraceutical, and medicinal industries, increasing the naturally occurring flavanone aglycones in fruit materials may generate new potential for utilizing these beneficial chemicals in various applications.

Grapefruit contains many nutrients and biologically active compounds. The results show that grapefruit’s albedo parts and segmental membranes have the highest total content of phenols, naringin, and naringenin. Grapefruit (*Citrus × paradisi* L.) extracts from the fresh juice and flavedo parts have greater levels of antiradical activity. These findings will be beneficial for processing grapefruit and creating products because there is a lack of literature on the phytochemical and taste qualities. According to the findings of this study, discarded fruit parts possess varied qualities and can also be employed to produce high-quality nutritional supplements and medicines.

## Figures and Tables

**Table 1:** Extract ID and preparation condition.

Extract ID *	Extraction Method	Solvent (Ethanol *v*/*v*)	Excipient (Cyclodextrins CD)
AT1 (control sample)	UAE combined with thermal hydrolysis	50%	
AT2 (control sample)		70%	
ST1 (control sample)		50%	
ST2 (control sample)		70%	
AA1		50%	α
AA2		70%	α
SA1		50%	α
SA2		70%	α
AB1		50%	β
AB2		70%	β
SB1		50%	β
SB2		70%	β
AG1		50%	γ
AG2		70%	γ
SG1		50%	γ
SG2		70%	γ

* First letter demonstrated fruit parts (A-albedo; S-segmental membrane); second letter (T-control sample, A-α-CD, B-β-CD, G-γ-CD); numbers (1–50% solvent *v*/*v*, 2–70% solvent *v*/*v*).

**Table 2:** The linearities of calibration curves of flavanones.

Component	Calibration Equation	Coefficient of Determination *R*^2^	Correlation Coefficient	LOD µg/mL	LOQ µg/mL
Naringin	Y = 25.50x + 6720	0.99992	0.99996	0.146	0.583
Naringenin	±Y = 33.30x + 3570	0.99992	0.99996	0.118	0.430
Narirutin	Y = 18.60x + 8100	0.99999	0.99999	0.281	0.5032

LOD—limit of detection; LOQ—limit of quantification.

**Table 3:** Yields of flavanones from control samples using ultrasound extraction method modified with thermal hydrolysis. Results are means ± SD (*n* = 3).

Extract ID	Naringin mg/g	Narirutin mg/g	Naringenin μg/g
AT1 *	13.97 ± 0.698	2.24 ± 0.12	23.58 ± 1.17
AT2 *	14.07 ± 0.70	2.36 ± 0.18	25.06 ± 1.25
ST1 *	10.53 ± 0.526	2.34 ± 0.17	65.84 ± 3.29
ST2 *	7.8 ± 0.39	1.95 ± 0.09	67.59 ± 3.37

* Extract ID are provided in Table 1. The highest amount of naringin and narirutin was determined from the albedo part 13.97 ± 0.698 mg/g, 14.07 ± 0.71 mg/g (50 and 70% ethanol *v*/*v*), and 2.24 ± 0.12 mg/g, 2.36 ± 0.12 mg/g (50 and 70% ethanol *v*/*v*), respectively. Meanwhile, naringenin’s highest quantity was detected from the segmental membrane—67.59 ± 2.81 µg/g using 70% of ethanol *v*/*v*.

**Table 4:** Flavanone concentrations retrieved from samples using CDs.

Extract ID *	Naringin mg/g	Narirutin mg/g	Naringenin µg/g
AT1	13.99	2.24	23.58
ST1	10.53	2.34	65.84
AA1	18.87	3.33	43.44
SA1	46.53	0.70	91.19
AB1	6.87	1.74	16.21
SB1	51.11	0.81	0.45
AG1	5.99	1.79	0.84
SG1	7.52	2.29	0
AT2	14.07	2.36	24.06
ST2	7.80	1.95	67.59
AA2	11.58	2.25	28.77
SA2	49.72	0.80	86.69
AB2	8.75	1.74	23.80
SB2	58.08	0.98	0.20
AG2	10.59	2.13	0
SG2	6.76	2.15	0.45

The quantitative yield of naringin, naringenin, and narirutin using (1%) additional components α-, β-, γ-CDs. Results are means ± SD (*n* = 3). Naringin–AA1 *p* < 0.05 AT1; SB1 *p* < 0.05 SA1, ST1; SB2 *p* < 0.05 SA2; AA1 *p* < 0.05 AA2; SB2 *p* < 0.05 SB1; narirutin–AA1 *p* < 0.05 AT1; naringenin–AA1 *p* < 0.05 AT1; SA1 *p* < 0.05 ST1; AA2 *p* < 0.05 AT2; SA1 *p* < 0.05 SA2. * Extract ID are provided in Table 1.

## Data Availability

Not applicable.
